# Medical documentation practice and associated factors among health workers at private hospitals in the Amhara region, Ethiopia 2021

**DOI:** 10.1186/s12913-022-07809-6

**Published:** 2022-04-09

**Authors:** Mulugeta Desalegn Kasaye, Miftah Abdella Beshir, Berhanu Fikadie Endehabtu, Binyam Tilahun, Habtamu Alganeh Guadie, Shekur Mohammed Awol, Mulugeta Hayelom Kalayou, Tesfahun Melese Yilma

**Affiliations:** 1 Department of Health Informatics, School of Public Health, College of Medicine and Health Sciences, Wollo University, Dessie, Ethiopia; 2 Department of Health Informatics, Institute of Public Health, College of Medicine and Health Sciences, University of Gondar, Gondar, Ethiopia; 3 Department of Health Informatics, School of Public Health, College of Medicine and Health Sciences, Bahirdar University, Bahir Dar, Ethiopia

**Keywords:** Medical documentation, Documentation practice, Record-keeping, Health workers, Private hospital, Amhara region

## Abstract

**Introduction:**

Medical documentation is an important part of the medical process as it is an essential way of communication within the health care system. However, medical documentation practice in the private sector is not well studied in Ethiopian context. The aim of this study was to assess the practice of medical documentation and its associated factors among health workers at private hospitals in the Amhara region, Ethiopia.

**Method:**

An institution-based cross-sectional quantitative study supplemented with a qualitative design was conducted among 419 health workers at the private hospitals in the Amhara Region, Ethiopia from March 29 to April 29 /2021. Data were collected using both a self-administered questionnaire and interview guide for quantitative and qualitative respectively. Data were entered using Epi data version 3.1 and analyzed using SPSS version 20. Descriptive statistics, Bi-variable, and multivariable logistic regression analysis were performed. In-depth interviews were conducted using semi-structured questionnaires with eight respondents to explore the challenges related to the practice of medical documentation. Respondent’s response were analyzed using OpenCode version 4.03 thematically.

**Results:**

Four hundred seven study participants returned the questionnaire. Nearly 50 % (47.2%) health workers had of good medical documentation practice. Health workers who received in-service training on medical documentation AOR = 2.77(95% CI: [1.49,5.14]), good knowledge AOR = 2.28 (95% CI: [1.34,3.89]), favorable attitude AOR = 1.78 (95%CI: [1.06,2.97]), strong motivation AOR = 3.49 (95% CI: [2.10,5.80]), available guide line formats AOR = 3.12 (95% CI: [1.41,6.84]), eHealth literacy AOR = 1.73(95% CI: [1.02,2.96]), younger age AOR = 2.64 (95% CI:[1.27,5.46]) were statistically associated with medical documentation.

**Conclusion:**

More than half of the medical services provided were not registered. Therefore, it is important to put extra efforts to improve documentation practice by providing planed trainings on standards of documentation to all health workers, creating positive attitudes and enhancing their knowledge by motivating them to develop a culture of information.

## Introduction

Medical documentation is an detail information or evidence for patients and a significant part of patient care due to medical reporting [1]. Documentation is a way of recording the account of what happened, when it happened and the act of authenticating with factual or substantial support of medical practices by qualified workers [2, 3]. In addition, medical documentation is the process of recording the plan of care, patients’ need, and interventions health providers need to implement and evaluation of the patient outcomes. Documentation includes a record of patient condition provided by health professionals; like clinical assessments, clinical evaluation, and professional judgment regarding the provision of patient care [4]. Specifically, it includes health documents, evaluation charts (a wall poster that help clinicians to track the progress of their patient), acts, tests (medical procedures involves testing sample of blood, urine and other substances from the human body), checklists (those formats used to collect data in an orderly and systematic manner), correspondence books, management reports, and clinical subjective notes or personal reflections; Physicians, nurses, midwives, laboratory technicians, Anesthesia and Pharmacy professionals can involve in medical documentation [1, 3]. High-quality medical documentation is expected in every area of care and every setting [5, 6]. Documenting the medical record of a patient is essential for continuity of patient care, research, legal defense, reimbursement and it enhances communication among healthcare providers [7], and patients concerning diagnoses, laboratory results, treatments, and outcomes [8]. To ensure the quality of medical services, good medical documentation is necessary [9], and the practice of keeping good medical records assists health workers to track, investigate and make informed decisions based on the medical profile of the patient easily. Medical records are believed to be the most vital resource that health workers use in their day-to-day practice in the health care arena. Health care facilities by-laws or policies should require medical staff members to complete patient records within the allotted time (allocated or specified work time) [10]. Whether it is paper-based or electronic, medical documentation should be patient-focused, accurate, relevant, clear, permanent, confidential, and timely [11].

On the other hand, poor medical documentation practice affects both patient management and medico-legal issues, which arise from a breakdown in communication resulting from incomplete and inadequate documents [12, 13]. Lack of e Health literacy, computer skill, and related training among health workers has an influence and is indicated as a barrier and challenging issue to the documentation of health related information [14, 15].

Despite studies globally identified limitations in medical documentation practice, keeping the medical record of the patient is part of a professional obligation [16, 17]. Poor medical care documentation is linked with avoidable medical errors that can lead to adverse patient outcomes [18]. A thumb rule of expression, “If it is not documented, it does not exist” holds true when we deal about documentation [19].

According to a survey by World Health Organization (WHO), poor communication between healthcare workers is one factor for medical errors [20]. Evidence in USA showed that approximately 1.3 million people are annually injured and at least one person is killed due to medical documentation and medication errors [21]. In Africa, health workers working in health care institutions experienced medical documentation record keeping as a major challenging practice facing several challenges that include lack of time to complete the record, and shortage of recording materials (tools that the health care providers used to document the information about the patient) [12]. Studies also identified that knowledge, attitude, in service trainings and practice shortages in medical documentation [22–26].

In Ethiopia, the act of medical documentation is poorly practiced and have reported left undone [2, 3]. The challenge for this could be due to lack of training, time, poor knowledge, and attitude of the health workers towards medical documentation [2, 27–29]. In spite of the limited amount of studies [2, 3, 30] done in Public Hospitals in Ethiopia, which is limited to nursing documentation practice among nurse workers, there is limited study has been indicated among Private hospitals that take the lion share of health care delivery to the population related to medical documentation practice and its associated factors. Private hospitals in Ethiopia has advantages for both the urban and rural communities by providing health care at any time when people are in need of particular services. Expanding private health sectors reduces the burden of the government as it increases consumer choice and competition [31]. Private hospitals are owned privately including for profits and non-profits. In reality, it is uncommon that good health service quality can be improved without recording the essential medical information about the patients. For good patient management and medical intervention, all the useful information of the patient should be well documented. Shortages in recording the medical history of the patients may resulted in miscommunications of health workers like nurses, physicians and others and this leads to adverse patient outcomes. However, the information generated by the health workers in private hospitals seems left unsearched and not clearly indicated. Hence, this study tried to revealing the magnitude of the problem and exploring the challenges for documenting the patient information by the health workers. The results of this study can be used as a benchmark for designing future studies by the upcoming researchers. The objective of this study was therefore, to assess the medical documentation practice and identifying the factors among health workers at private hospitals in the Amhara region, Ethiopia.

## Methods

### Study design and setting

An institution-based cross-sectional quantitative study supplemented with qualitative design was conducted at all Private hospitals in the Amhara region, Ethiopia, from March 29–April 29, 2021.

The Amhara region is located in the Northwestern and North Central parts of Ethiopia. It has 13 administrative zones, 1 special zone, 181 woredas, and 78 urban centers. Amharic is the working language of the state. The capital city of the State of Amhara is Bahir-Dar. There are seven referral public hospitals in the Region. There are ten private hospitals, with approximately 699 health workers working in the private hospitals, in the Amhara Region. These hospitals serve for inpatient and outpatient services. Each private hospital has a minimum of 50 beds to serve. They serve as a referral centers for the local community at time of emergency.

### Study population

The source population for this study were all health workers working in private hospitals in the Amhara Region. We included 699 health workers working at the private hospitals and who were available during the data collection period were interviewed. The quantitative study design was supplemented with qualitative to explore challenges related to the practice of medical documentation in the hospitals. Data were collected from March 29 to April 29 / 2021.

### Sample size determination

The sample size was determined using single proportion formula taking the proportion of medical documentation practice 45.4%, from a study conducted previously at University of Gondar Hospital [32], 95% confidence interval CI, 5% margin of error. The final sample was 419 by taking 10% non-response rate. There are ten private hospitals in the Amhara region. From each hospital, permanent health workers working at the hospital during the study period are included in this study; proportional allocation was done for each hospital based on the actual number. The proportionate number of study subjects was determined by using, *n* = nf/N*ni Where ni = the number of workers in each hospital, nf = total sample size, *N* = total number of health workers working in Amhara region private hospitals. Each health worker from each private hospital was selected using a simple random sampling method from the administrative health workers’ list of records. All health workers working in private hospitals who were permanent workers and available during data collection period were included in the study.

A structured and self-administered questionnaire was developed to collect data regarding medical documentation practice and its associated factors. An English version questionnaire was used. The questions were developed based on the national guideline prepared by the FMOH (EHRIG) and different survey tools that had previously been pre-tested [2, 3, 17, 30, 33, 34]. Trainings were given for data collectors and supervisors to keep the data quality issue.

Prior to the actual data collection period, the items were pretested at Woldiya general hospital with 5% (21 samples) of the total health workers in the hospital. The results were used to check reliability and consistency of questionnaire and some modifications were made accordingly. Cronbach alpha was used to check the reliability of the questionnaire and its value was 0.84.

Qualitative data was obtained by in depth interviews of health workers that includes physicians, nurses, midwife and matrons who were purposively selected considering saturation of ideas. A semi-structured questionnaire and appropriate sound recorder was used to conduct the in-depth interviews. At the beginning, the sample size for the qualitative method was not determined. Instead, the interview continued until the idea redundancy has occurred. A total of eight [8] health professionals were interviewed. The principal investigator, (Mulugeta Desalegn Kasaye), transcribed and translated the data.

### Operational definitions

#### Medical documentation practice

The practice by which medical records are written by health workers on patient status, medical care, and medical care responses including evaluations, medical diagnoses, planned care, medical interventions or treatments delivered, patient education, and interdisciplinary communication. So, one can use the term medical record like a paper-based record, manually written documents, health records, and the likes. The medical documentation practice of study participants was measured using 11 “Yes”, and “No” questions items. For analysis, the “Yes” item was coded as “1” whereas the “No” item was coded as “0”. By taking the 75th percentile value as a cutoff point the level of practice was good if the score was above and poor if the score was below [3].

#### Knowledge towards medical documentation

The knowledge of study participants was measured using 10 ‘Yes’ and ‘No’ questions. The level of knowledge was categorized as Good level and Poor level by taking the mean score of knowledge questions. A score above the mean value was represented as “Good knowledge”, whereas “Poor knowledge” below the mean score [3].

#### Attitude towards medical documentation

The level of attitude was measured using 14 Likert scale questions with five responses ranging from “strongly agree [5]” to “strongly disagree [1]” and then the level of attitude was determined based on the median score. If the participants scored above or equal to the median score of attitude questions then they were categorized as having a favorable attitude else unfavorable towards medical documentation practice [35].

#### Good knowledge towards medical documentation

Respondents who scored above the mean score of knowledge questions [3].

#### Favorable attitude towards medical documentation

Those respondents who scored above the median score of attitude questions [35].

#### Health workers

In this study health, workers were workers who had direct contact with patients, took patient data through direct assessment, and who used and reused the past-recorded patient records for the dent of their timely activity. Those included medical doctors, nurses, midwives, Laboratory technicians, and others in the context of this study.

### Statistical analysis

Data were entered manually using Epi Data Version 3.1 and exported to SPSS version 20 for analysis. Descriptive statistics were performed. Binary logistic regression used to identify factors associated with Medical documentation practice. Variables with *p*-value < 0.2 in binary logistic regression fitted to multivariable logistic regression. *P*-values of less than 0.05 at multivariable logistic regression were set to declare statistical significance. Adjusted odds ratio (AOR) and 95% CI used to determine the strength of association.

For the qualitative method, the data, which were collected through in-depth interview, and it was analyzed thematically. The in-depth interview was conducted in Amharic. Data were transcribed to Amharic and later translated into English. The translated data were coded, categorized, and synthesized using Open Code version 4.03. The data was coded into three themes and quotes from each theme were used for the result. Interview questions were directed towards three categories of investigation: medical documentation practice Feelings (Purposes), challenges of medical documentation practice, initiatives (Solutions) of medical documentation practice.

## Results

### Socio-demographic characteristics

Out of 419 distributed questionnaires, 407 responses received with a response rate of 97.13%. Among the study participants, 212 (52.1%) were females. The mean age of the respondents was 30 (SD = ±6.58) years with the majority of the age group were from 25 to 29 years. In terms of the marital status of the respondents, the majority 205(50.4%) were married. Regarding educational status, the majority 199 (48.9) were Bachelor of Science (BSc) Degree holders (Table 1).

**Table 1:** Socio demographic characteristics of health workers working in Private Hospitals in the Amhara Region, March 2021 (*n* = 407)

Variable	Category	Frequency (407)	%
Age	20–24	72	17.7
	25–29	169	41.5
	30–35	110	27.0
	36 and above	56	13.8
Sex	Male	195	47.9
	Female	212	52.1
Marital Status	Single	185	45.5
	Married	205	50.4
	Widowed	8	2.0
	Divorced	7	1.7
	Separated	2	0.5
Educational level	Diploma	138	33.9
	Bachelor of Science	199	48.9
	Medical Doctor	40	9.8
	Specialty	30	7.4
Monthly salary	< 6193	245	60.2
	6194–7071	26	6.4
	7072–8017	31	7.6
	8018–9056	22	5.4
	Above 9057	83	20.4
Type of Profession	Nurse	191	46.9
	Midwife	60	14.7
	Doctor	70	17.2
	Laboratory	66	16.2
	Anesthesia	7	1.7
	Pharmacy	13	3.2
Work setting	Inpatient	228	56.0
	Outpatient	179	44.0

### Health workers attitude towards medical documentation practice

The respondents attitude score was summed and dichotomized in to two based on the median attitudes score which was 40 (S.D = ±7.5). More than half 235 (57.7%) of the respondents with [95%CI: 52.5, 62.4] had a favorable attitude towards medical documentation practice. Most, 247 (60.7%) of the study respondents strongly agree on medical documentation has an advantage in the day-to-day activities and 244 (55.0%) of the respondents strongly agree on medical documentation has a positive impact on patient safety.

### Health workers knowledge towards medical documentation practice

The score of respondents’ knowledge toward medical documentation practice was added up and dichotomized into two based on the mean knowledge score which was seven (S.D = ± 2.04). Based on this cut-off point, health workers with good knowledge on good medical documentation practice found to be 246 (60.4%) with [95% CI: 55.4, 65.6]. Most of the study participants 369 (90.7%) knew an identified activity (about the patient) should be documented. More than two-third 291 (71.5%) of the participants knew the education provided to the patient must be documented in the medical record. About 345(84.8%) of the study participants knew documentation of patients care is part of professional responsibility.

### Health workers motivation towards medical documentation practice

Health workers motivation was assessed using 10 Yes and No questions. By adding the 10 Yes/No item questions, the result was then dichotomized holding the median value 8 with S.D. = ±2.17. The score above the median, categorized as having Strong Motivation else Weak Motivation. The median score used as a measure of central tendency for the sake of this study; hence, the distribution of the data was not normal. In this study, 207 (50.9%) of the participants had Strong Motivation towards Medical Documentation Practice with [95% CI: 46.2, 56.5]. The majority 353 (86.7%) of the study participants were eager to have a good patient record in every condition. From the study participants, 318 (78.1%) try to record with the other health staff because it makes the work run more smoothly. Two-third of the participants showed that their supervisors were aware of how medical recordings are going at the work.

### Health workers computer skill towards medical documentation practice

The computer skill of health workers was assessed using a five-point Likert scale questions ranging from (Never = one, rarely = 2, Weekly = 3, Daily = 4, and Several times a day = 5). By summing the values of each question and by taking the median 15 (S.D = ±6.3) value of the result, two categories were created as Good Computer skill above or equal to the median and the remaining Poor Computer skill if below the median Value. Health workers scoring above were categorized with Good computer skill towards medical documentation practice. Of all the study participants, 212 (52.1%) of them had good Computer skill with [95%CI: 48.0, 57.2] and the rest had poor computer skill towards medical documentation practice. Most 117(27.3%) of the respondents were capable of searching for health-related information online daily. A small number 20(4.9%) of Health workers had been questioned by patients about online means of contacting them.

### E-Health literacy level of health workers toward medical documentation practice

Two hundred twelve (52.1%) of the respondents with 95% CI of 47.4 to 57.2 of respondents had scored above or equal to the median [36] (S.D = ±8.6) value and had high eHealth Literacy. The majority 160 (39.3%) of the respondents agreed they feel the internet is useful in helping them in decision-making about their health. Similarly, most 185 (45.5%) of the respondents agreed that they know how to use the health information they find on the Internet to help themselves. Around 128 (31.4%) of the respondents strongly agreed that they know how to use the Internet to answer their questions about health.

### The practice of health workers towards medical documentation practice

To measure the level of medical documentation practice of health workers 10 Yes and No questions were used and the scores were added up and then dicatomized in to two as Good and Por. Accordingly, the level of medical documentation practice was measured by the 75th percentile cutoff of point. A score greater than or equal to the 75th percentile was classified as having good medical documentation practice, otherwise as poor. Of the total participants, one hundred ninety-two (47.2%) with a 95% CI of 41.8 to 52.0 of respondents had scored above or equal to the 75th percentile value and had good medical documentation practice. Among all respondents, 385 (94.6%) attach the medical notes to the patient chart after they complete it. The majority 381(93.6%) of the respondents completely document the medical diagnosis of patients. Around 302 (74.2%) of the respondents document medication allergies and adverse reactions prominently in the record. Most 287 (70.5%) of the study participants voluntarily reported any medical errors that occur while providing medical care to their leaders.

### Organizational factors

This study sought to discover organizational factors, which could affect the medical documentation practice of health workers. A very small 80 (19.7%) number of respondents were received in-service training on medical documentation. From the total 80 health workers who received in-service training, 38 (60%) of them had taken the training on medical documentation just 2 years back. Around 246 (60.4%) of the health workers were providing medical care for more than twenty patients per day. More than half, 253 (62.16%), of the health workers were witnessed the availability of standard sheets in their working unit. Most 278 (68.4%) of the respondents were familiar with the medical documentation standards (Table 2).

**Table 2:** Organizational Factors of Health Workers working at Private hospitals in the Amhara Region, Ethiopia, 2021

Variables	Response	Frequency	Percentage (%)
Have ever trained on MDP (*n* = 407)	Yes	80	19.7
	No	327	80.3
When did you receive training (*n* = 80)	<=2 years ago	32	40
	> 2 years ago	48	60
The average number of patients seen by a single profession per day (*n* = 407)	< 20 patients	161	39.6
	> = 20 patients	246	60.4
Availability of standard sheets (*n* = 407)	Yes	253	62.16
	No	154	37.84
Familiar with medical documentation standards (*n* = 407)	Yes	278	68.4
	No	129	31.6
Availability of medical care plan materials (guidelines) (*n* = 407)	Yes	345	84.8
	No	62	15.2

**Fig. 1:**
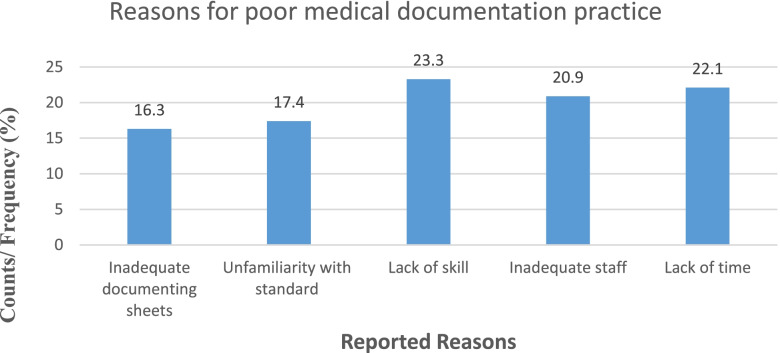
Reasons for poor medical documentation practice

Respondents were also asked the reasons for not documenting all the medical care provided to the patient and around 20 (23.3%) and 19 (22.1%) of them reported that lack of skill and lack of time respectively hampered their medical documentation practice. Other reasons such as inadequate documenting sheet, 14 (16.3%), unfamiliarity with standard sheet, 15 (17.4%) and inadequate staff, 18 (20.9%) were also mentioned for documentation of medical care (Fig. 1).

### Factor associated with medical documentation practice of health workers

Binary and multivariable logistic regression analyses was conducted to assess the statistical associations between independent variables and the dependent variable, medical documentation practice. The variables assessed in the bivariable logistic regression analysis include Age, Sex, work setting, in-service training, knowledge, attitude, motivation, eHealth literacy, availability of guide lines, availability of medical sheet and computer skill were statistically associated with medical documentation practice at *P* value less than 0.2. However, multivariable logistic regression was done to control the potential confounders. Accordingly, age, knowledge, attitude, in-service training motivation, eHealth literacy, and availability of guidelines were still significant at a *P*-value of 0.05.

Accordingly, Health workers whose age belongs to 25–29 AOR (1.27, 5.46) were 2.65 times more likely to document than their counterparts do (age 20–24 years). Health workers who had good knowledge AOR (1.34, 3.89) were 2.17 times more likely to have good medical documentation practice than those who had poor knowledge. Health workers who had Favorable Attitude AOR (1.06, 2.97) were 1.78 times more likely to have good medical documentation practice than those who had an Unfavorable attitude. The odds of good medical documentation practice among trained AOR (1.49, 5.14) health workers were 2.77 times higher than those who did not trained. Health workers who had strong motivation AOR (2.10, 5.80) were 3.49 times more likely to have good documentation practice than those who had weak motivation. Health workers who used the available guideline formats AOR (1.41, 6.84) in the hospital were 3.12 times more likely to have good medical documentation practice than those who did not use it. Compared to those health workers who had low eHealth literacy, those who had high eHealth literacy AOR (1.02, 2.96) were 1.73 times more likely to have good documentation practice (Table 3).

**Table 3:** Bivariable and multivariable logistic regression analysis of factors associated with medical documentation practice among health workers at Private hospitals, Amhara, Ethiopia, 2021 (*n* = 407)

Variable	Category	Practice Level *n* = 407)		COR (95%)	AOR (95%)
		Poor (n, %)	Good (n, %)		
Sex	Male	93(47.7)	102(52.3)	1.49(1.01,2.20)	1.06(0.63,1.79)
	Female	122(57.5)	90(42.5)	1	1
Age	20–24	53(73.6)	19(26.4)	1	1
	25–29	84(49.7)	85(50.3)	2.82(1.54,5.17)^a^	2.64(1.27,5.46)^b^
	29–35	51(46.4)	59(53.6)	3.23(1.69,6.15)^a^	2.17(0.97,4.85)
	36 and above	27(48.2)	29(51.8)	2.99(1.43,6.29)	1.83(0.74,4.52)
Work Setting	Inpatient	132(57.9)	96(42.1)	1.60(1.07,2.36)^a^	1.23(0.74,2.03)
	Out patient	83(46.4)	96(53.6)	1	1
Knowledge	Poor	116(72.0)	45(28.0)	1	1
	Good	99(40.2)	147(59.8)	3.83(2.49,5.87)^a^	2.28(1.34,3.89)^b^
Attitude	Unfavorable	112(65.1)	60(34.9)	1	1
	Favorable	103(43.8)	132(56.2)	2.39(1.59,3.59)^a^	1.78(1.06,2.97)^b^
Motivation	Weak	147(73.5)	53(26.5)	1	1
	Strong	68(32.9)	139(67.1)	5.67(3.70,8.69)^a^	3.49(2.10,5.80)^b^
In-service Training	Not Trained	188(57.5)	139(42.5)	1	1
	Trained	27(33.8)	53(66.2)	2.65(1.59,4.43)^a^	2.77(1.49,5.14)^b^
Availability of Medical sheet	No	100(64.9)	54(35.1)	1	1
	Yes	115(45.5)	138(54.5)	2.22(147,3.36)^a^	1.50(0.89,2.55)
Availability of Guideline formats	No	49(79.0)	13(21.0)	1	1
	Yes	166(48.1)	179(51.9)	4.06(2.13,7.76)^a^	3.12(1.41,6.84)^b^
eHealth Literacy	Low	128(65.6)	67(34.4)	1	1
	High	87(41.0)	125(59.0)	2.75(1.84,4.11)^a^	1.73(1.02,2.96)^b^
Computer skill	Poor	119(61.0)	76(39.0)	1	1
	Good	96(45.3)	116(54.7)	1.89(1.28,2.89)^a^	1.12(0.65,1.94)

*Note*: *P* –Value ≤0.2 for Bi-variable analysis *P*- Value ≤0.05 for multi variable analysis, a Factors associated with medical documentation practice in bi-variable analysis, b Factors associated with medical documentation practice in multivariable analysis, 1 Reference

### Interview quotations for each key theme

Eight in depth interviews were conducted among health works. The health workers had responsibilities and firsthand information regarding medical documentation practice in the hospital. The two health workers were matron of the hospital. The other two were OPD team coordinator general physicians and the remaining four nurse workers were interviewed. Seven of the respondents were males. The median age and work experience of the respondents were 32 range [3, 24–35, 37, 38] and 9 years range [2–20] respectively.

### Theme: medical documentation activity feelings

Health workers were asked how they about the medical documentation process and their activity. Most of the interviewed health workers agreed that the importance of documenting the health information of the patient though there is poor medical documentation practice among health workers in the health facilities and they felt the work is not satisfying.

“...*A medical documentation is a process of describing a patient’s life history and is based on the information provided by the patient and done by a qualified health professional. Nevertheless, usually it seems unimportant part of work by which workers left it without reasoning*”. (Respondent 8).

Health professionals stated that though they tried to document the patient information using manual recording system, the activity would be hampered due to nature of the manual recording system.

“*… The current documentation practice is not satisfying yet because by its nature paper based recording system is tiresome”. (*Respondent 2).

Another nurse professional who disclosed his best experience in medical documentation activities and its importance as a functional requirement for health providers in documenting the medical history of the patient/clients.

“… *The medical documentation is a must and is very important to be done. In public health facility the documentation is done timely and simultaneously with the care rendered. However, in private hospitals medical documentation is done and practice as additional work. This is because; the number of health workers and the shift is not proportional so that documentation will be done after the service has been delivered to that particular patient.*”” (Respondent 5).

Participants mentioned their feeling regarding medical documentation; as if documented patient information is valuable, the care providers were not usually capable of doing it on time.

*“… The documentation practices helps the work done worthless. If it is not documented, you will loss the value of your work. I think every professional knows the importance of information. However, there are weaknesses in the documentation process. The reason for these weaknesses is the inability to document services provided to the patient on time. There is a problem of not documenting health information of the patient, thinking that I will do it later.”* (Respondent 4).

### Theme: challenges related to the practice of medical documentation among health workers

The major gaps in relation to the medical documentation practice among health workers were lack of trainings, supervision and feedback, shortage of documenting materials, lack of computerized system, poor knowledge and the habit of delaying tasks.

“… *There are some things in the hospital that are not enough. For example, Stool Request and Urine Request forms may not regularly available. The technician refrains from registering the results of the investigations until these tools are printed. This leads the patient not be able to receive services on time.”* (Respondent 8).

A general physician who was an Outpatient department (OPD) team leader said:

“*While I check (audit) the medical cards, I got undocumented patient information in these files. That is because there is no documentation habit by the staff.” (*Respondent 3*).*

A nurse professional working at emergency department in hospital said:

*“There is a top-down gap in medical documentation practice both at the leadership level and as an individual professional. There are people who always want to be pressured to do documentation. The higher body does not receive much attention. Only if the professional reports the case is acceptable. The top body does not recognize all of the information and only accepts the report, does not care about the facts listed and documented”.* (Respondent 6).

Health professionals dictated that though good medical documentation promotes patient safety and quality of care, complete and accurate medical record keeping hampered by lack of formats and documentation sheets in the working units.

***“…*** *The formats to be printed are found only in a single office that in case neglects me to document the patient data. Printers are not available in each department so that lack of documentation sheets are one of the challenges to be considered to not document the client information****” (***Respondent 3).

### Theme: solutions for poor documentation practice

Health workers had commented their best solution for the betterment and effectiveness of the medical documentation practice and the means to solve the barriers. Conducting trainings on medical documentation, motivating staffs, building a positive attitude and increasing the availability of standard documenting materials would enhance the practice of documenting the health information of patients/ clients.

An important strategy to solve the problems resulted from health professionals when dealing with medical documentation is the need for training. Training would minimized the knowledge gaps of the health workers as it boosts the culture of documentation by enhancing the performance of the service providers.

“… *Training is needed to increase the knowledge of workers regarding medical data assessment and documentation*. *I am happy if special training is given to workers on medical documentation*. *For effective medical documentation practice professional training should be provided from time to time. Training will enhance the professional qualification and motivation.” (*Respondent 3, Respondent 6*).*

Participants described that their daily patient information gathering depends up on the documentation sheets, which are developed based on the standard that the hospital adopted from the national standard for documenting patient data, and that enhance the work done with simple and user-friendly manner.

*“… Adaptations to Standard documentation sheets are very important in our day-to-day activities. We use them currently and will be used in the future in our hospital. The use of these standard sheets may decrease medical malpractice and the work done will be fast as much as possible.” (*Respondent 1*).*

The participants has stated that a positive attitude of individual health care provider would have paramount importance for documenting all the profiles of the patient. The value of the medical information greatly witnessed the work that has been done by that particular health care provider.

*“… Documentation is an important part of professional life. Undocumented medical information is valueless. If not all medical information are recorded, I will not be able to treat the patient properly, I will not be able to provide the information properly if s/he needs it, and Medco Legal will hold me accountable if there are any. If I have a positive attitude towards medical documents, my work is worth more. Therefore, building a positive attitude will solve problems regarding medical documentation of my customers.”* (Respondent 6).

Participants put their comment about the necessity of regular monitoring of each providers service that has been given for their clients by their supervisors. Those assigned coordinators should regularly monitor and check the patient cards immediately after follow up of the patient.

“… *Hospital coordinators should regularly monitor the coordinating unit to ensure that any unregistered medical information is available through contact with stakeholders. It is important to check whether the patient’s card is being written before returning the card to the card room.*” (Respondent 8).

Health workers also indicated the use of computerized or electronic system in the hospitals would simplify the work regarding medical documentation of the patient profile. Despite its unavailability, computers can solve problems like losing of patient information, unreadability, and it enhance searching or retrieving of each patient information using unique identifier easily.

***“…*** *It is obvious that the medical documentation work has been done using paper forms from the past 15 years up to now. In a near future, we will use an all in one system, which will be computerized. Every hospitals must be connected via computers and the works performed will be much easy as a result. Referral systems will be in advance because of computerized system workflow.*” ***(***Respondent 4).

Participants were elaborated the use of electronic medical recording system in the hospital would enhance the health professionals work more easily and solve the documentation problems.

*“… If all the departments of the hospital, from the card room to the pharmacy, are equipped with a computer system, the documentation problem can be solved. I think it would be good to start with these systems in the future, as things that fill up a computer require everything and cannot be left undocumented.” (*Respondent 7)

## Discussion

This cross-sectional study aimed to assess medical documentation practice and associated factors among health workers working at private hospitals in the Amhara region, Ethiopia. The findings of this study showed that good attitude, knowledge, motivation, availability of guideline formats, as well as high eHealth literacy level and in service training had a significant effect on medical documentation practice.

Previous conducted evidences found that medical documentation practice is one of the important medical care activities that are left undone [3, 36–38]. For this study, self-reported documentation practice was used to determine factors associated with medical documentation practice among health workers working at Private Hospitals in the Amhara Region, Ethiopia.

In this study, the level of medical documentation practice was found to be poor, 47.2%(*n* = 192) [95% CI (41.8, 57.8)]. The result was consistent with a study done in Tigray [30] where the level of documentation practice was inadequate, England 47% [39], Canada 53.3% [40], and University of Gondar hospital 45.4% [32]. However, this finding was higher from Indonesia 37% [41], University of Gondar hospital (37.4%) [3]. The discrepancy might be due to the number of study samples selected. Our study sample size was much higher than these studies. The other possible reason might be due to the study setting, in these studies the study settings were single site (department based i.e. conducted only in wards) but this study covers both inpatient and outpatient departments in a multisite based. In contradict with this, high (98%) satisfactory level of documentation practice was reported in Jamaica hospital [42]. The possible reason for this discrepancy might be due to care providers were familiar with the guidelines prepared in each ward and the respondents adequate knowledge as the study revealed [43]. Most (52.8%) of the study participants revealed poor medical documentation practice which corresponds with a study conducted in Felegehiwot referral hospital [44], where medication errors were administered due to bad medical documentations. This finding is lower than the finding from South Africa, cape town [45]. This could be due to poor knowledge of the care givers about the necessity of the medical documentation [46].

For this study, self-reported medical documentation practice was assessed to identify factors associated with medical documentation practice among health workers working at private hospitals. In the current study, health workers who had good knowledge regarding medical documentation are more likely to have good documentation practice as compared to those health workers who had poor knowledge. This study is in line with the studies from Nigeria [43], Iran [47], Jamaica [25], Gondar [3], Dire Dewa [35]. The possible reason for this might be good knowledge of medical documentation improves familiarity with documentation guidelines and enhances medical documentation practice as a responsible professional.

Health workers who had a favorable attitude towards medical documentation were more likely to have good documentation practice compared to those health workers who had unfavorable attitude. This insight is consistent with study results in Uganda [23], Dire Dewa [35], Hawassa [28], Tigray [30], and University of Gondar Hospital [3]. This could be because of having a favorable attitude towards medical documentation increases the motivation of service providers to document the medical care they delivered and the other possible reason might be the learning habit of workers regarding documentation will appreciate the value of medical documentation as honorable health personnel.

In-service training on the standard medical care processes was significantly associated with good medical documentation practice. Health workers who received in-service training were more likely to have good medical documentation practice as compared to those who did not trained. This is in line with studies done in Ghana [24], Uganda [23], Dire Dewa [35], and Gondar [3]. This might be because of training increases knowledge to use the medical documentation guideline formats and familiarity of standard documentation tools and it enhances the likelihood of grasping positive attitudes to document the cares provided to the patient/client. It is believed that pertaining proper training should increase the knowledge of the health workers so that good medical documentation practice, as a result would improve.

Moreover, in this study, we tried to assess the motivation of health workers towards medical documentation practice. Hence, health workers who had strong motivation were more likely to have good medical documentation practice as compared to those who had weak motivation. This finding is consistent with findings from Dire Dewa [35], this might be due to the fact that motivated workers will develop a positive attitude so that the documentation practice will be done responsibly. However, poorly motivated workers may loosely perform his/her works.

Availability of standardized guidelines had a positive association with medical documentation practice. This finding has a matching effect with a study conducted in Australia [26], Western Jamaica [25]. This might be because of effective medical practice is supported by the availability of proper and standardized guidelines. The other possible reason for this could be; the accuracy and quality of medical documentation is highly integrated with the availability of standardized working guidelines in the medical home [29].

In this study, eHealth literacy had a positive association with medical documentation practice among health workers. Those health workers with high eHealth Literacy were more likely to have good medical documentation practice than those who had low eHealth literacy. This might be due to the need for information is depending on the quality of information that has already been registered/ documented. The other possible reason for this could be having good eHealth literacy improves in locating, using, and evaluating online health information [15]. This to be realized important health information should be documented online and this improves the health care providers’ access to that information when a need arises.

In this study, age had a positive significant association with medical documentation practice. This finding contradicts with studies conducted in Dire Dewa [35], and Iraq [48] where older health workers (age above 30 years had a positive association with medical documentation practice). The possible explanation for this could be the current private hospitals has a tendency to recruit younger health professionals. Recent graduated health professionals were available to work in private hospitals since the public hospitals are set to be saturated to recruit new graduates.

Furthermore, this study tried to identify different self-reported barriers to the medical documentation practice of health workers. The participants stated that lack of skill, lack of time, inadequate staff, unfamiliarity with standard documentation sheets, and inadequate documentation sheets as potential impediments for documentation. In line with this finding, studies from Nigeria [49] and Tigray [30] reported lack of time, inadequate documentation sheets as a major barrier to medical documentation among health workers .

### Limitation of the study

The main limitation of the study is that it did not include the patient record, which is a representative measure of the documentation practice for each health professional. Furthermore, as this study was used self-administered questionnaire most of the variables might have been exposed to social desirability bias. However, we recruited data collectors outside of the study hospitals (who were not members of the study hospitals), and we believed that we minimized the bias to our maximum effort. In addition, we cannot able to make comparisons for the findings due to shortages of comparable studies conducted to this area.

## Conclusion

This study finding offer valuable information about medical documentation practice and barriers of health workers working at private hospitals in the Amhara region. In this study, the overall medical documentation practice at private hospitals in the Amhara Region was poor. Inadequate documenting sheets, unfamiliarity with standard sheet, lack of skill, inadequate staff, and lack of time were the reasons for poor medical documentation practice. Having good knowledge, attitude, motivation, in-service training on medical documentation practice as well as the availability of guideline formats, higher eHealth literacy level, and younger age contributes to good medical documentation practice.

Enhancing the knowledge and attitude of health workers towards documentation of both the planned and provided medical care is high value in to improve medical documentation practice.

Efforts need to be made to conduct in service-trainings on medical documentation sfor all health workers without regarding any specifications. Therefore, this fosters the medical documentation practice knowledge, attitude, and motivation of the workers. Redesign-friendly guideline formats should also be the focus area by the federal ministry of health to make all medical guideline formats consistent, specific, and clear. Matrons of the private hospitals should be dedicative and able to create good knowledge to the team members frequently to establish a well and profound documentation system. Leaders of the private hospitals should motivate the staff to produce quality documentation. . We also recommend the upcoming researchers to study the medical documentation practice of health workers using observation method to know and evaluate the representativeness of the patient record.

## Data Availability

The data sets generated and/ analyzed in the current study are not available publicly due to the consent we took from the study participants in order not to share this data to others but are available from the corresponding author up on a reasonable request.

## Abbreviations

AOR: Adjusted odds ratio
CI: Confidence interval
MDP: Medical documentation practice
SD: Standard deviation
SPSS: Statistical package for social science
USA: United States of America
WHO: World health organization
